# Pilates in the Workplace: A Systematic Review and Meta-Analysis of Physical, Psychological, and Functional Outcomes in Employees

**DOI:** 10.3390/jfmk11030340

**Published:** 2026-08-29

**Authors:** Ioannis Tsartsapakis, Ioannis Trigonis, Aglaia Zafeiroudi, Evgenia Kouli, Maria Gerou

**Affiliations:** 1 Department of Physical Education & Sport Science, Aristotle University of Thessaloniki, 62122 Serres, Greece; miamixan@hotmail.com; 2 Department of Physical Education & Sport Science, Democritus University of Thrace, 69100 Komotini, Greece; itrigon@phyed.duth.gr (I.T.); ekouli@phyed.duth.gr (E.K.); 3 Department of Physical Education & Sport Science, University of Thessaly, 42100 Trikala, Greece; azafeiroudi@uth.gr

**Keywords:** Pilates method, workplace health promotion, meta-analysis, occupational health, musculoskeletal disorders, functional disability

## Abstract

**Background**: Work-related musculoskeletal disorders impose substantial global burdens. Although Pilates is a recognized biopsychosocial intervention, a quantitative synthesis evaluating its occupational efficacy remains limited. **Objective**: To compute the pooled effect sizes of workplace Pilates interventions on musculoskeletal pain intensity, functional disability, muscular endurance, and health-related quality of life (QoL). **Methods**: Twelve randomized controlled trials (14 reports) involving diverse occupational cohorts were analyzed. Data dependency was resolved via outcome aggregation. Random-effects models calculated pooled effect sizes (Hedges’ *g*), 95% confidence intervals (CI), and 95% prediction intervals (PI). Risk of bias was assessed using RoB 2. **Results**: The meta-analysis demonstrated a moderate, statistically significant reduction in functional disability (*g* = −0.660, 95% CI [−1.091, −0.229], *p* = 0.011). Conversely, pooled estimates for pain intensity (*g* = −0.461, *p* = 0.360), muscular endurance (*g* = 1.354, *p* = 0.223), and QoL (*g* = −0.146, *p* = 0.815) were non-significant. Substantial heterogeneity was observed (*I*^2^: 32.02–98.25%), and all prediction intervals crossed the null, indicating high uncertainty regarding future effects. **Conclusions**: Workplace Pilates consistently mitigates functional disability, but current evidence does not support significant pooled improvements in absolute pain, endurance, or QoL. This divergence between functional recovery and pain reduction aligns with biopsychosocial frameworks, suggesting improved work capacity despite variable pain relief. Given the high heterogeneity and wide prediction intervals, findings must be interpreted cautiously, highlighting the need for rigorously standardized trials.

## 1. Introduction

Work-related musculoskeletal disorders (WMSDs) represent a significant public health and socioeconomic challenge, causing economic burdens, physical discomfort, and functional limitations across the global workforce. Recent epidemiological data indicate a high prevalence of WMSDs across diverse occupational settings on a global and continental scale. Broadly, global incident musculoskeletal disorder cases rose from 211.80 million to 334.74 million between 1990 and 2017 [1]. Specifically, recent worldwide systematic reviews and meta-analyses highlight a severe burden in healthcare environments. For instance, a global meta-analysis among nurses reported a pooled WMSD prevalence of 81.1%, identifying the low back (58.5%), neck (45.9%), and shoulder (40.9%) as the most commonly affected body regions [2]. Continent-level data further substantiate these findings, demonstrating high overall prevalence among nurses in Europe (61.3%) and the Americas (59.3%) [3], as well as among surgeons, where prevalence peaks in Asia (77.6%) and Europe (73.1%) [4]. In addition, specific 12-month prevalence rates among clinical personnel remain notably high, ranging from 64.2% to 78.2% [5,6]. Similarly elevated rates are well-documented among sedentary groups, such as academic staff [7] and office-bound teleworkers facing specific ergonomic hazards [8], as well as specialized occupational cohorts subjected to unique vehicle vibrations and prolonged asymmetric driving or flight postures. These conditions negatively impact work capacity and health-related quality of life [9,10].

To mitigate musculoskeletal pain and functional limitations, workplace physical activity and active exercise interventions are increasingly utilized [11,12]. Within the context of active occupational rehabilitation, the Pilates method has received attention for its emphasis on core stabilization, controlled movement patterns, and neuromuscular control. Primary trials and systematic reviews have supported its utility in broader clinical populations, demonstrating efficacy in reducing pain scores and improving functional capacity for individuals experiencing chronic non-specific low back pain [13,14,15] and chronic neck pain [16].

Beyond localized pain reduction, structured Pilates interventions support physiological adaptations, including enhanced trunk muscle endurance, improved proprioception, and optimized spinal alignment [17,18]. Furthermore, contemporary occupational medicine recognizes that work-related musculoskeletal pain often coexists with psychological strain and occupational stress. Accordingly, Pilates has been studied as a mind–body intervention capable of addressing both somatic and psychological dimensions [19]. Recent evidence suggests that Pilates protocols may positively influence mental health indices and quality of life parameters in employees [20].

Despite the expanding literature on workplace health promotion, establishing guidelines for the optimal implementation of workplace Pilates remains complex. Previous systematic reviews have mapped the qualitative evidence, suggesting that the method functions as a biopsychosocial intervention [21]. However, these prior reviews were primarily descriptive. Due to anticipated clinical and methodological heterogeneity across diverse occupational cohorts, they refrained from pooling quantitative findings. Consequently, a gap remains in the literature: despite the growing number of workplace Pilates trials, there is a lack of quantitative synthesis evaluating its effects across occupational cohorts. The pooled effect sizes for key employee outcomes have not yet been established.

To address this gap, the present meta-analysis synthesizes quantitative data from randomized controlled trials (RCTs) implemented in occupational environments. By focusing on extractable outcomes from these settings, this study provides pooled effect estimates for musculoskeletal pain intensity, functional disability, muscular endurance, and health-related quality of life. Through this quantitative synthesis, the review aims to move beyond narrative summaries and provide a data-driven foundation to inform occupational health practices and corporate wellness policies.

## 2. Materials and Methods

### 2.1. Study Design and Guidelines

This meta-analysis was conducted in strict accordance with the Preferred Reporting Items for Systematic Reviews and Meta-Analyses (PRISMA 2020) guidelines [22]. Structural alignment and adherence to the formal reporting standards were validated via the PRISMA 2020 Checklist, which is available for review in Supplementary File S1 (Section S2). The review protocol was prospectively registered in the International Prospective Register of Systematic Reviews (PROSPERO; registration ID: CRD420261439523).

### 2.2. Literature Search and Information Sources

A comprehensive and systematic literature search was executed across four primary electronic databases from inception to 6 July 2026: PubMed, Scopus, the Cochrane Central Register of Controlled Trials (CENTRAL), and the Physiotherapy Evidence Database (PEDro). No language or publication date restrictions were applied. To capture grey literature and ongoing studies, trial registries including ClinicalTrials.gov and the World Health Organization International Clinical Trials Registry Platform (ICTRP) were systematically searched, complemented by targeted searches on Google Scholar. The review protocol was prospectively registered on PROSPERO (CRD420261439523).

The search string was constructed using a combination of Medical Subject Headings (MeSH) terms and free-text keywords, including but not limited to: “Pilates”, “workplace”, “occupational health”, “employees”, “workers”, “musculoskeletal pain”, “functional disability”, and “randomized controlled trial”. The primary objective of this search was to identify randomized and controlled trials suitable for quantitative meta-analytic synthesis, expanding upon and distinctively quantifying the conceptual framework outlined in recent qualitative systematic literature evaluations [21].

The comprehensive electronic search architecture, including the precise Boolean search strings and filters applied across all screened databases, is fully documented in Supplementary File S1 (Section S1).

Study selection was conducted in duplicate and independently by two reviewers (I.Ts. and I.Tr.), who screened all titles, abstracts, and full-text articles against predefined eligibility criteria. Any discrepancies between reviewers during the screening phase were resolved through structured consensus discussion or, when necessary, via final adjudication by a third senior reviewer (A.Z.). Inter-rater reliability for full-text study inclusion demonstrated high agreement (Cohen’s kappa = 0.88).

### 2.3. Eligibility Criteria (PICOS Framework)

Studies were selected for inclusion in this meta-analysis based on pre-specified criteria adhering to the PICOS framework.

For the Population (P), we included active employees and occupational populations across various professional settings (e.g., office workers, healthcare and nursing personnel, industrial workers, and aviation or helicopter pilots) experiencing work-related musculoskeletal disorders or symptoms, as well as healthy working cohorts.

The Intervention (I) consisted of structured Pilates-based exercise programs, irrespective of the delivery mode (mat-based, equipment-based, or apparatus Pilates), duration, frequency, or setting (on-site workplace or off-site sessions).

The Comparison (C) involved passive control groups (e.g., no intervention, waiting list, usual care) or active control groups (e.g., ergonomic education, general physical activity, alternative exercise modalities).

To be eligible for quantitative synthesis, the Outcomes (O) required studies to provide sufficient statistical data (means, standard deviations, and sample sizes) to calculate effect sizes for at least one of the following target outcomes: functional disability, muscle endurance, health-related quality of life, or musculoskeletal pain intensity.

Finally, regarding the Study Design (S), eligibility was strictly limited to randomized controlled trials (RCTs) providing extractable pre- and post-intervention quantitative data.

### 2.4. Data Extraction and Study Quality

Two reviewers (I.Ts. and I.Tr.) independently performed data extraction, capturing key study characteristics (author, year, country, occupation, sample size), intervention details (frequency, volume, duration), and pre- versus post-intervention outcome metrics (means and standard deviations).

Methodological quality and risk of bias were independently assessed by two reviewers (I.T. and E.K.) using the standard Cochrane Risk of Bias tool for randomized trials (RoB 2). Disagreements during the quality assessment phase were resolved through consultation with a third reviewer. To ensure that only methodologically sound trials contributed to the quantitative synthesis, studies assessed as having an overall “High Risk of Bias” were strictly excluded from the meta-analysis (though no eligible studies in this review met this exclusion criterion).

Across the included trials, a total of 582 participants contributed data to the quantitative synthesis. The detailed baseline configurations, occupational characteristics, and intervention protocols extracted from the 12 included randomized controlled trials are structured within the comprehensive repository data framework provided in Supplementary File S1 (Section S3).

### 2.5. Statistical Analysis

Quantitative data synthesis and meta-analytic estimations were performed using JASP software (Version 0.97.0) [23]. To account for the dependency of data arising from multiple effect sizes within the same study cohorts, we applied the Outcome Aggregation method in JASP. This ensured that each study contributed only a single, aggregated effect size per outcome category, thereby resolving the issue of statistical dependency. To account for potential sample size imbalances and mitigate small-sample bias across the included trials, effect sizes were calculated and expressed as Hedges’ g along with their corresponding 95% Confidence Intervals (CIs).

Given the anticipated clinical and methodological diversity regarding occupational demographics, workplace environments, and Pilates intervention parameters, a random-effects model was systematically applied for all analyses. The Restricted Maximum Likelihood (REML) estimation method was utilized to calculate between-study variance (tau-squared). Furthermore, to account for the uncertainty in the estimation of residual heterogeneity and to control for Type I error rates, the conservative Knapp-Hartung adjustment was applied to all pooled effect sizes, adjusting the test statistics using a t-distribution.

Statistical heterogeneity among the included studies was evaluated using Cochrane’s *Q* chi-squared test and quantified via the I-squared statistic. Heterogeneity thresholds were defined as low (less than or equal to 25%), moderate (25% to 50%), and high (greater than 50%). Statistical significance for the pooled effect sizes was established at *p* < 0.05. Publication bias was evaluated through visual inspection of residual funnel plots (standard errors versus residuals). Formal quantitative tests for asymmetry (e.g., Egger’s regression test) were omitted, as standard methodological guidelines (e.g., Cochrane Handbook) explicitly advise against test-based asymmetry assessments when the number of available studies is small (*k* ≤ 6) due to insufficient statistical power.

To evaluate the robustness of the pooled effect estimates and to identify potentially influential studies, a systematic leave-one-out (one-study-removed) sensitivity analysis was performed for each primary outcome. The procedure was implemented through the Casewise Diagnostics function in JASP, which recalculates heterogeneity parameters (τ, τ^2^, *Q*_e_) after omitting each study. Influence metrics (standardized residuals, Cook’s distance, DFFITS, covariance ratios, and hat values) were examined to determine whether any single trial disproportionately affected the pooled estimates or the variance structure.

### 2.6. Effect Size Calculation

Effect sizes were calculated as Hedges’ g using strictly post-intervention means and standard deviations. Because the eligibility criteria were restricted solely to randomized controlled trials, baseline comparability between groups was generally assumed and confirmed upon methodological inspection of the included studies. Furthermore, change scores or ANCOVA-adjusted estimates were not consistently reported across all trials. Therefore, to ensure methodological consistency and strictly avoid mixing different effect size metrics within the same model, only post-intervention data were utilized for the quantitative synthesis. The pooled standard deviation was computed as follows:

$$SD_{\mathrm{pooled}}=\sqrt{\frac{(n_{E}-1)SD_{E}^{2}+(n_{C}-1)SD_{C}^{2}}{n_{E}+n_{C}-2}}$$

To correct for small-sample bias, the standard correction factor J was applied:

$$J=1-\frac{3}{4(n_{E}+n_{C})-9}$$

The effect size was then calculated as:

$$g=J\cdot \frac{M_{exp}-M_{ctrl}}{SD_{\mathrm{pooled}}}$$

The standard error of *g* was computed using:

$$SE=\sqrt{\frac{n_{E}+n_{C}}{n_{E}n_{C}}+\frac{g^{2}}{2(n_{E}+n_{C}-2)}}$$

To ensure clear interpretation, a consistent effect-direction coding rule was applied across outcomes without reversing measurement scales prior to pooling. For clinical outcomes where lower scores denote clinical improvement (i.e., pain intensity and functional disability), negative effect sizes (*g* < 0) indicate a reduction in symptoms favoring the Pilates intervention. Conversely, for outcomes where higher scores reflect desirable clinical adaptations (i.e., muscular endurance and health-related quality of life), positive effect sizes (*g* > 0) indicate improvements favoring Pilates.

## 3. Results

### 3.1. Literature Flow and Study Selection

The initial database search yielded a total of 432 records (PubMed: *n* = 144, Scopus: *n* = 211, PEDro: *n* = 32, Cochrane Library: *n* = 45). Following the removal of 114 duplicate entries, 318 records underwent title and abstract screening. Of these, 245 records were excluded. Out of the remaining 73 reports sought for retrieval, 2 reports could not be successfully obtained.

**Figure 1:**
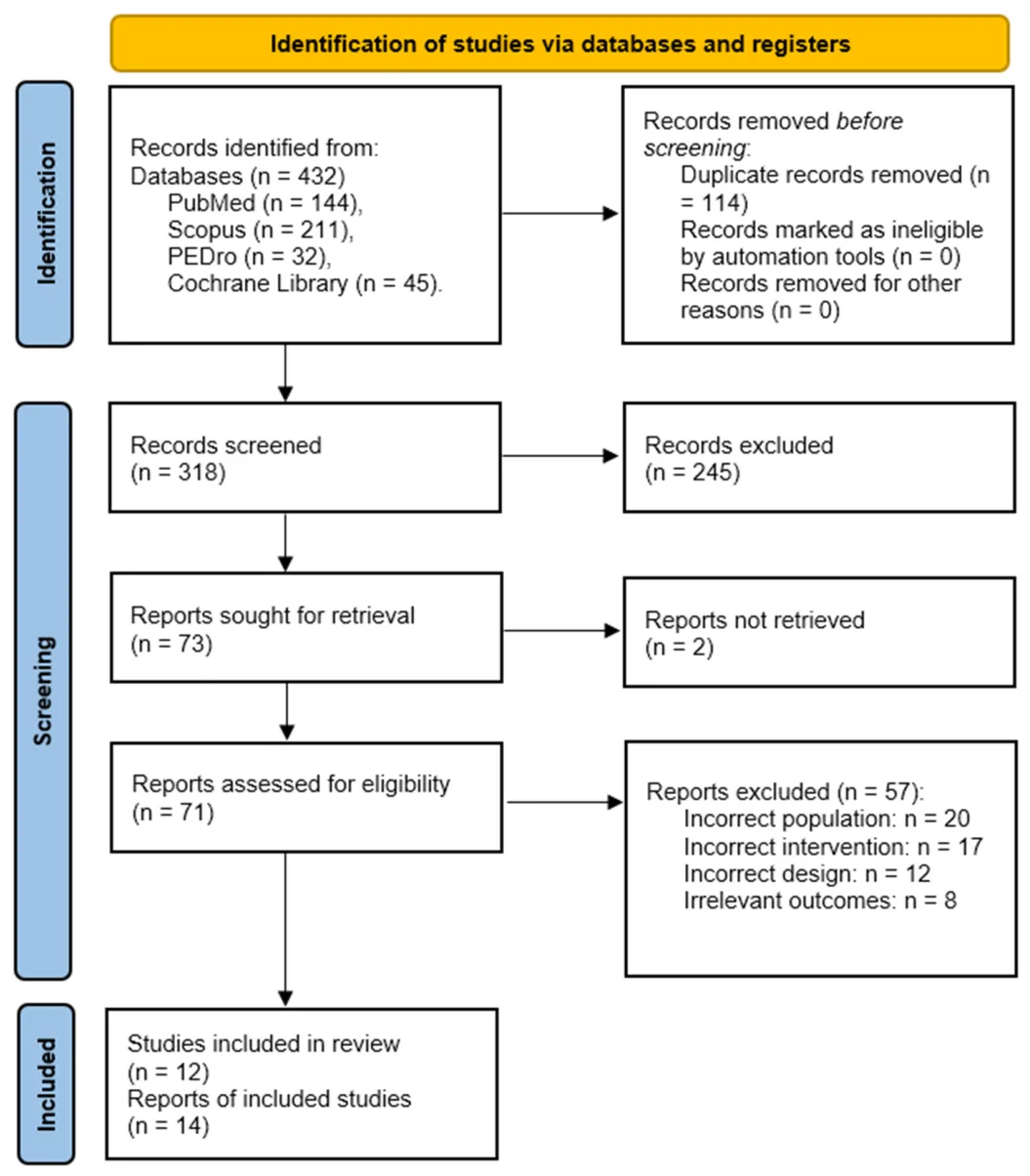
PRISMA 2020 Flow Diagram.

Consequently, 71 full-text reports were assessed for eligibility. A total of 57 reports were subsequently excluded based on explicit criteria: incorrect population (*n* = 20), incorrect intervention (*n* = 17), incorrect study design (*n* = 12), and irrelevant outcomes (*n* = 8). Ultimately, 14 individual reports, corresponding to 12 distinct randomized controlled trials, met all criteria and were included in the quantitative meta-analysis. The selection process is mapped in the PRISMA flow diagram (Figure 1).

### 3.2. Characteristics of Included Studies

The quantitative synthesis included 12 distinct trials, sourced from 14 published reports. The investigated populations comprised office workers and healthcare/nursing personnel, and military helicopter pilots. The characteristics of the included trials are summarized in Table 1, detailing the target populations, sample sizes, intervention protocols, and extracted outcomes.

### 3.3. Risk of Bias Assessment

Methodological quality and risk of bias were evaluated using the Cochrane RoB 2 tool. The 12 included trials demonstrated a low to moderate risk of bias (Some Concerns) across the evaluated domains. The detailed domain-level judgments for each randomized controlled trial are presented in Table 2, providing a comprehensive overview of the risk of bias assessments across all RoB 2 domains.

### 3.4. Meta-Analytic Outcomes

#### 3.4.1. Pain Intensity

To ensure clarity in the interpretation of the pooled estimates, effect sizes (Hedges’ *g*) were coded according to the clinical direction of the outcome measures. Specifically, for pain intensity and functional disability, negative effect sizes indicate a reduction in symptoms, thereby favoring the Pilates intervention. Conversely, for muscular endurance and health-related quality of life, positive effect sizes represent an improvement in performance or well-being, which also favors the Pilates intervention. This coding rule was consistently applied to all extracted data prior to aggregation.

The meta-analysis for musculoskeletal pain intensity incorporated *k* = 7 aggregated effect sizes. The pooled effect size for subjective pain intensity did not achieve statistical significance, presenting an estimate of Hedges’ *g* = −0.461 (95% *CI*: −1.599 to 0.677, *SE* = 0.465, *p* = 0.360). Statistical heterogeneity was high and significant (Cochran’s *Q* = 48.83, *df* = 6, *p* < 0.001, *I*^2^ = 93.03%, tau^2^ = 1.214, tau = 1.102). The 95% prediction interval ranged from −3.387 to 2.465. The summary statistics for the aggregated random-effects model, including the pooled effect size, heterogeneity indices, and prediction interval, are presented in Table 3.

**Figure 2:**
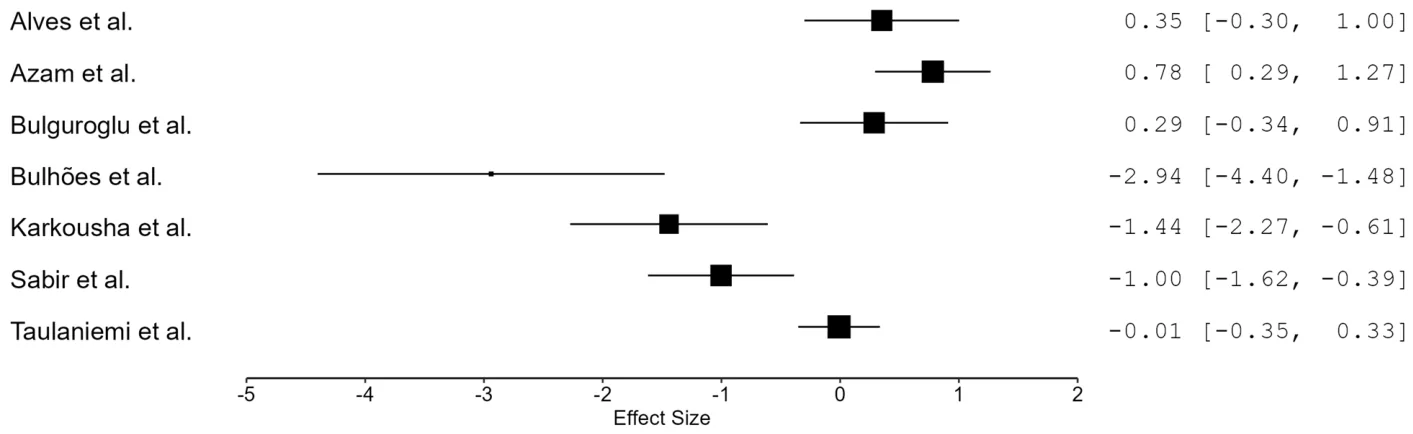
Pain Intensity Forest Plot. The forest plot illustrates the aggregated effect sizes and corresponding 95% confidence intervals for all included trials assessing musculoskeletal pain intensity: Alves et al. [30] Azam et al. [28], Bulguroglu et al. [32], Bulhões et al. [35], Karkousha et al. [25], Sabir et al. [24], and Taulaniemi et al. [34]. Note: The ordering of studies from top to bottom follows an alphabetical sequence. Effect sizes are presented as Hedges’ *g* with their respective 95% confidence intervals.

The forest plot in Figure 2 illustrates the aggregated study-level effect sizes and confidence intervals for all included trials assessing musculoskeletal pain intensity.

**Figure 3:**
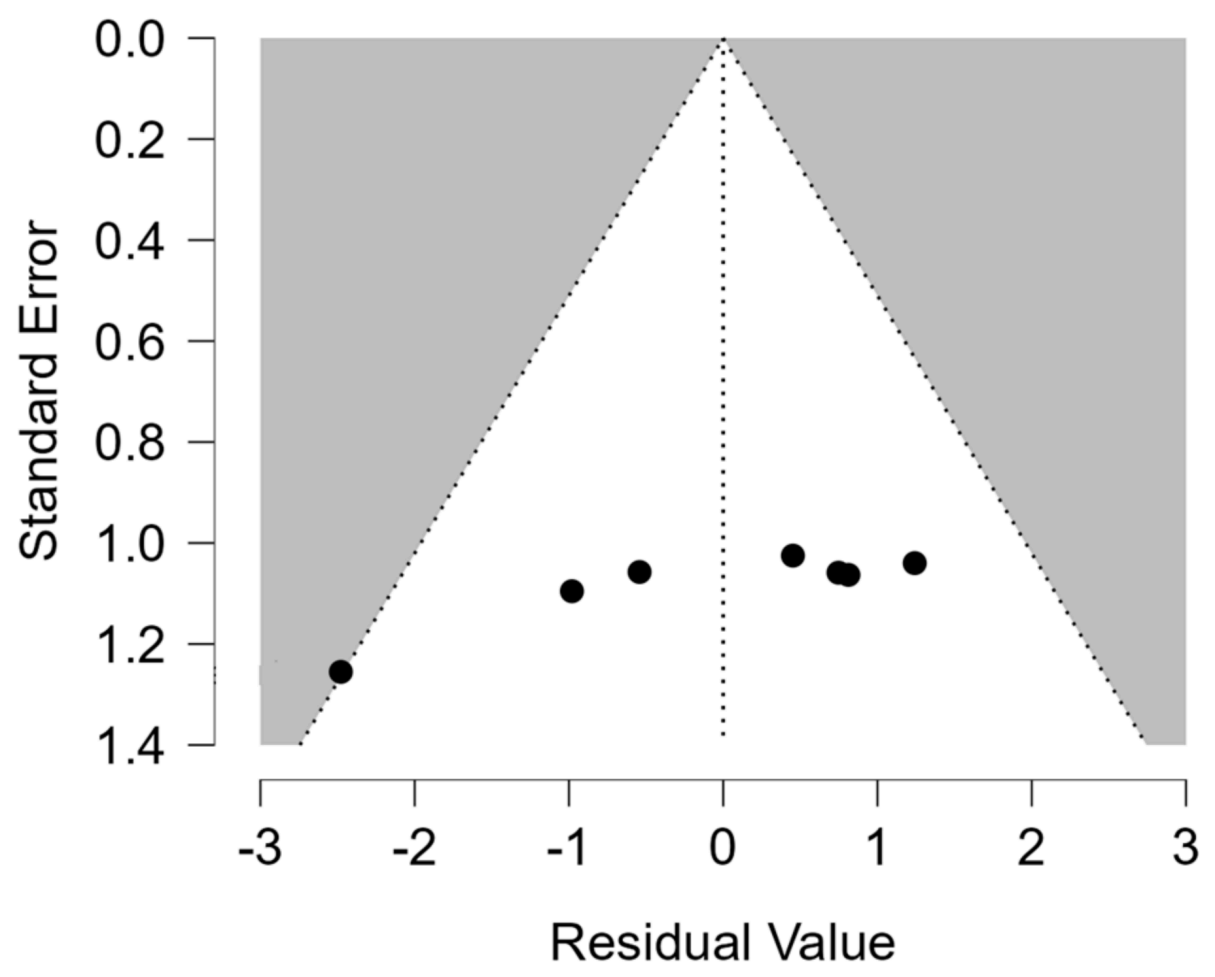
Pain Intensity Residual Funnel Plot. The funnel plot displays residual values against standard errors under an aggregated random-effects model. Note: The vertical dashed line represents the pooled effect, dots denote individual studies, and the grey shaded area outlines the pseudo 95% confidence region.

The funnel plot in Figure 3 presents the distribution of the aggregated effect sizes for pain outcomes to visually assess potential publication bias and small study effects.

#### 3.4.2. Functional Disability

The meta-analysis for functional disability incorporated *k* = 6 aggregated effect sizes. The aggregated random-effects model indicated a statistically significant pooled effect size of Hedges’ *g* = −0.660 (95% CI: −1.091 to −0.229, SE = 0.168, *p* = 0.011, *t*(5) = −3.94). Statistical heterogeneity was not significant (*Q* = 7.14, *df* = 5, *p* = 0.211, *I*^2^ = 32.02%, 95% CI: 0.00% to 87.61%, tau^2^ = 0.057, tau = 0.239). The 95% prediction interval ranged from −1.409 to 0.089. The summary statistics for the aggregated random-effects model examining functional disability are presented in Table 4, including the pooled effect size, heterogeneity indices, and prediction interval.

**Figure 4:**
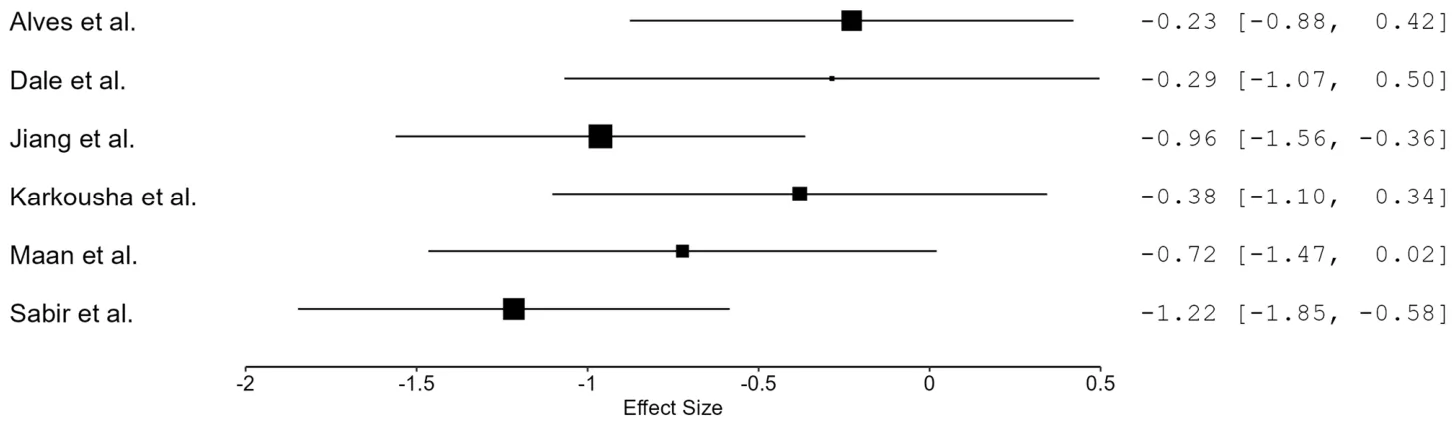
Forest plot illustrating the aggregated effect sizes for functional disability across the included randomized controlled trials under an aggregated random-effects model: Alves et al. [30], Dale et al. [31], Jiang et al. [33], Karkousha et al. [25], Maan et al. [27], and Sabir et al. [24]. Note: The ordering of studies from top to bottom follows an alphabetical sequence. Effect sizes are presented as Hedges’ *g* with corresponding 95% confidence intervals.

The forest plot in Figure 4 displays the aggregated study-level effect sizes and confidence intervals for all included trials evaluating functional disability.

**Figure 5:**
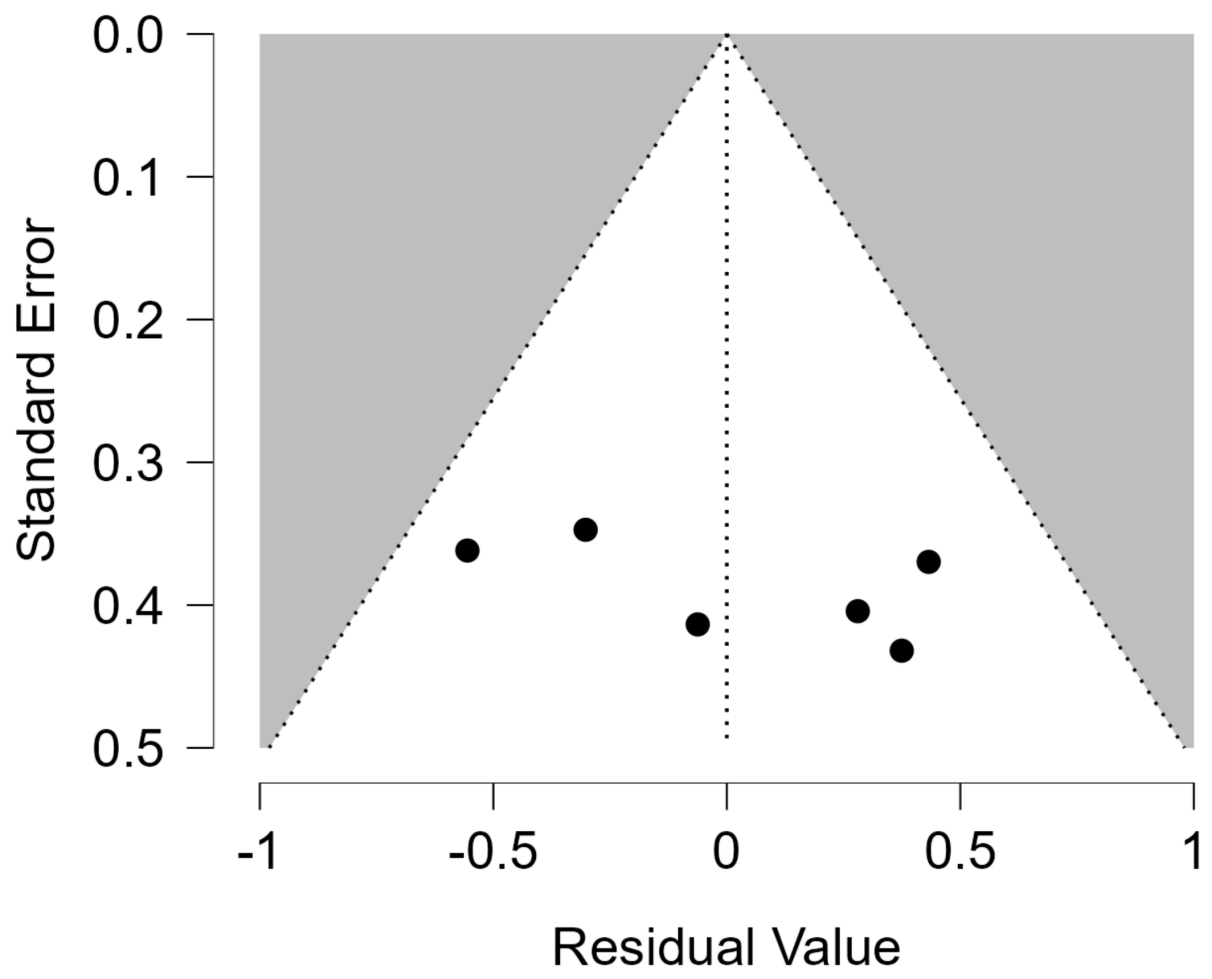
Funnel plot of residual values and standard errors for functional disability outcomes under an aggregated random-effects model. Note: Residual values are plotted against standard errors to evaluate small study effects.

The funnel plot in Figure 5 provides a visual assessment of potential publication bias and small study effects for the aggregated functional disability outcomes.

#### 3.4.3. Muscular Endurance

The meta-analysis for muscular endurance incorporated *k* = 6 aggregated effect sizes. The aggregated random-effects model indicated a non-significant pooled effect size of Hedges’ *g* = 1.354 (95% CI: −1.150 to 3.857, *SE* = 0.974, *p* = 0.223, *t*(5) = 1.39). Statistical heterogeneity was extreme and significant (Cochran’s *Q* = 104.57, df = 5, *p* < 0.001, *I*^2^ = 97.54%, 95% CI: 93.49% to 99.61%, tau^2^ = 5.383, tau = 2.320). The wide 95% prediction interval crossed the line of no effect, ranging from −5.115 to 7.822. The summary statistics for the aggregated random-effects model examining muscular endurance are presented in Table 5.

**Figure 6:**
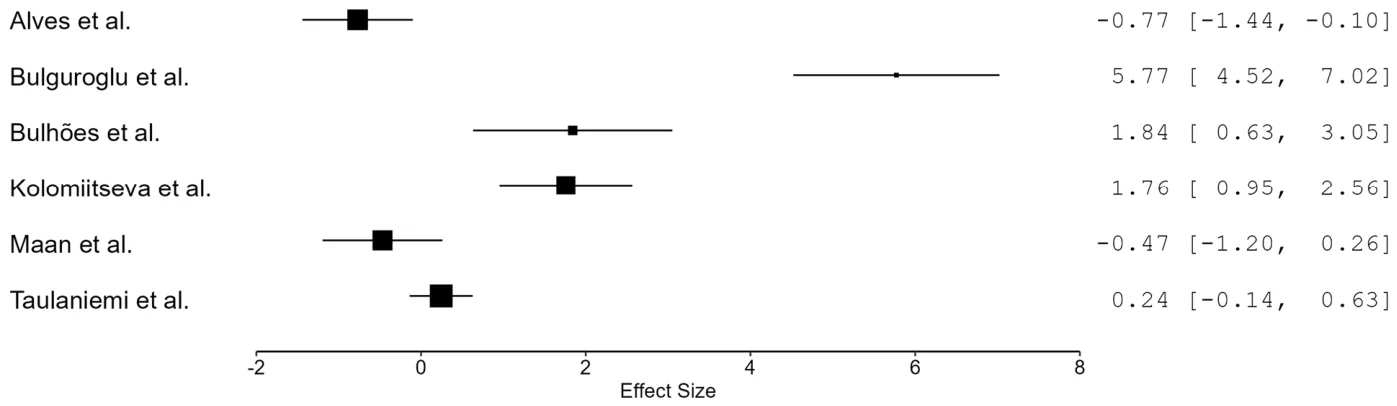
Forest plot illustrating the aggregated effect sizes for muscular endurance across the included randomized controlled trials under an aggregated random-effects model: Alves et al. [30], Bulguroglu et al. [32], Bulhões et al. [35], Kolomiitseva et al. [26], Maan et al. [27], and Taulaniemi et al. [34]. Note: The ordering of studies from top to bottom follows an alphabetical sequence. Effect sizes are presented as Hedges’ g with corresponding 95% confidence intervals.

The forest plot in Figure 6 displays the aggregated study-level effect sizes and confidence intervals for all included trials evaluating muscular endurance.

**Figure 7:**
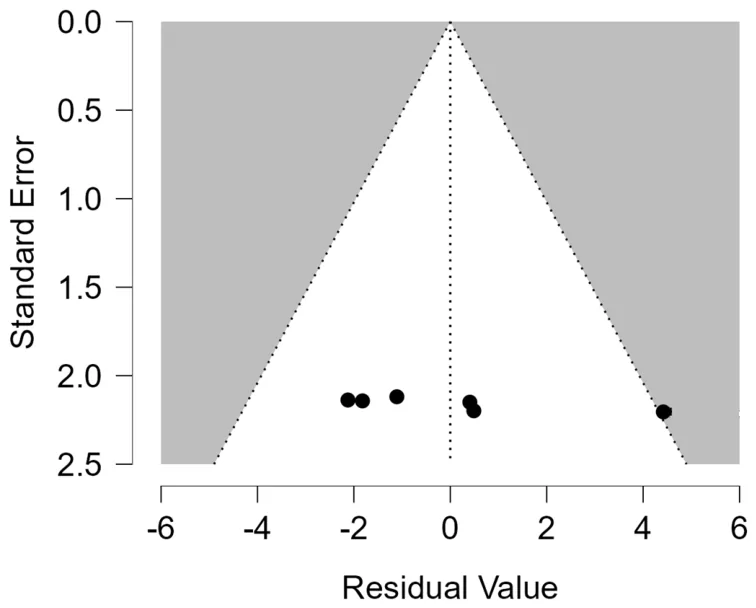
Funnel plot of residual values and standard errors for muscular endurance outcomes under an aggregated random-effects model. Note: Residual values are plotted against standard errors to evaluate small study effects.

The funnel plot in Figure 7 provides a visual assessment of potential publication bias and small study effects for the aggregated muscular endurance outcomes.

#### 3.4.4. Quality of Life

The meta-analysis for health-related quality of life incorporated *k* = 2 aggregated effect sizes. The aggregated random-effects model indicated a non-significant pooled effect size of Hedges’ *g* = −0.146 (95% CI: −6.338 to 6.046, SE = 0.487, *p* = 0.815, *t*(1) = −0.30). Statistical heterogeneity remained significant (*Q*(1) = 9.41, *p* = 0.002, *I*^2^ = 89.37%, 95% CI: 46.61% to 99.95%, tau^2^ = 0.425, tau = 0.652). The 95% prediction interval was wide and crossed the line of no effect, ranging from −10.491 to 10.190. The summary statistics for the aggregated random-effects model examining health-related quality of life are presented in Table 6.

**Figure 8:**
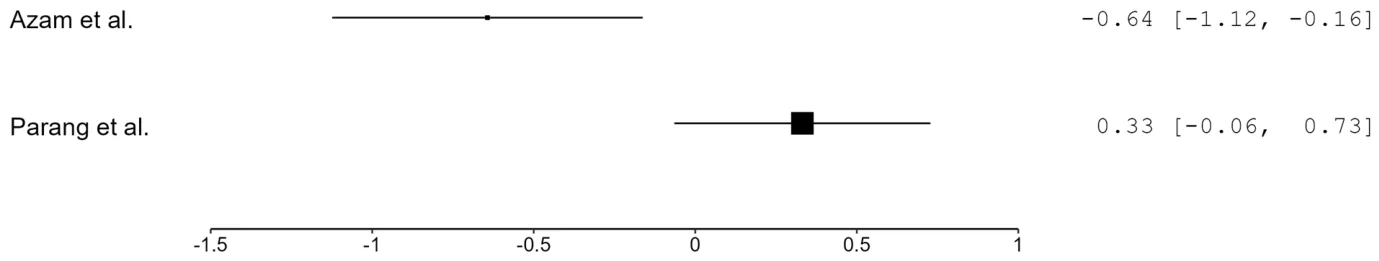
Forest plot illustrating the aggregated effect sizes for health-related quality of life across the included randomized controlled trials under an aggregated random-effects model: Azam et al. [28] and Parang et al. [29]. Note: The ordering of studies from top to bottom follows an alphabetical sequence. Effect sizes are presented as Hedges’ *g* with corresponding 95% confidence intervals.

The forest plot in Figure 8 displays the aggregated study-level effect sizes and confidence intervals for all included trials evaluating health-related quality of life.

**Figure 9:**
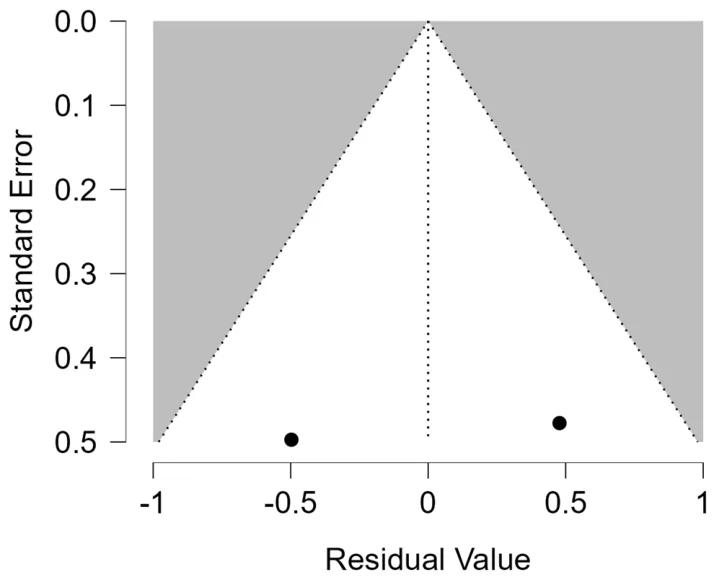
Funnel plot of residual values and standard errors for health-related quality of life outcomes under an aggregated random-effects model. Note: Residual values are plotted against standard errors to evaluate small study effects. Due to the small number of studies (*k* = 2), symmetry evaluation is limited.

The funnel plot in Figure 9 provides a visual assessment of potential publication bias and small study effects for the aggregated quality-of-life outcomes.

#### 3.4.5. Certainty of Evidence (GRADE Assessment)

A formal certainty-of-evidence assessment was conducted for each primary outcome using the Grading of Recommendations Assessment, Development and Evaluation (GRADE) framework (Table 7). Overall, the certainty of evidence was rated as Low for functional disability and Very Low for musculoskeletal pain intensity, muscular endurance, and health-related quality of life.

The primary reasons for downgrading included:

a. Risk of bias: Performance and response bias inherent to non-blinded exercise trials.
b. Inconsistency: Unexplained substantial statistical heterogeneity (*I*^2^ > 89%) observed in pain, endurance, and quality of life.
c. Imprecision: Small sample sizes per outcome (k ≤ 7) and wide 95% prediction intervals crossing the line of no effect.

### 3.5. Sensitivity Analyses

Following the outcome aggregation strategy to resolve unit-of-analysis errors, the number of independent studies per outcome category was substantially reduced (ranging from *k* = 3 to *k* = 6). Due to this small number of included studies per model, traditional leave-one-out sensitivity analyses and robust casewise diagnostics (e.g., Cook’s distance; see Supplementary Materials, Section S4) were deemed mathematically unstable and of limited diagnostic value. Consequently, systematic study-level exclusions were not formally interpreted, as removing even a single aggregate effect size from such small pools would artificially distort the between-study variance (tau^2^) and fail to provide meaningful inferences about model robustness. The wide prediction intervals and persistent high heterogeneity (*I*^2^) observed in the primary analyses accurately reflect the current uncertainty within this specific evidence base.

## 4. Discussion

### 4.1. Summary of Main Findings and Meta-Analytic Synthesis

This meta-analysis synthesized quantitative evidence from randomized controlled trials evaluating workplace-based Pilates interventions for musculoskeletal pain intensity, functional disability, muscular endurance, and health-related quality of life. After resolving unit-of-analysis dependencies, the pooled estimates revealed a highly heterogeneous pattern of effects.

The most consistent finding was a moderate, statistically significant reduction in functional disability among employees participating in Pilates programs. In contrast, pooled estimates for musculoskeletal pain intensity, muscular endurance, and health-related quality of life were not statistically significant. Although individual trials reported gains in these areas, the aggregated synthesis showed high statistical heterogeneity (*I*^2^ ranging from 89.4% to 97.5%). This variability likely reflects substantial differences in occupational settings, intervention formats, and baseline characteristics across the employee cohorts (e.g., sedentary office workers versus healthcare personnel).

Collectively, these findings suggest that while workplace Pilates interventions may not uniformly enhance all facets of physical performance and subjective well-being, they consistently reduce functional disability. The lack of significant improvement in pain metrics, contrasted with the improved functional capacity, highlights a complex relationship between symptom perception and actual physical functioning in the workplace.

### 4.2. Psychosocial Effects of Pilates

The psychosocial benefits of Pilates are well-documented in broader populations, with systematic reviews and trials reporting improvements in quality of life, mood, and stress regulation [20,38,39,40,41]. However, our meta-analytic findings in occupational settings present a more nuanced picture. Although previous literature highlights gains in quality of life, our pooled estimate for health-related quality of life was not statistically significant and showed high heterogeneity.

This discrepancy suggests that the psychosocial benefits of Pilates may be context-dependent in active workforces. Modern workplaces involve cognitive loads, organizational stressors, and physical fatigue that might attenuate the effects of exercise interventions. While our aggregated data did not demonstrate a uniform improvement in quality of life, broader literature indicates Pilates may still support positive psychological constructs like mindfulness and life satisfaction through its mind–body approach [20,42].

Outcomes related to fatigue and specific psychological symptoms often vary across studies [40,43,44]. Overall, while Pilates is generally considered a psychosocially beneficial exercise, its effects on quality of life in the workplace are less definitive. This variability likely stems from differences in occupational stressors, baseline psychological health, and intervention protocols, suggesting that psychosocial gains may require tailored program designs.

### 4.3. Biomechanical Mechanisms and Physical Adaptations

Although our pooled analysis did not show a statistically significant improvement in muscular endurance, the biomechanical principles of Pilates help explain the observed reductions in functional disability. Pilates emphasizes controlled movement and the activation of deep stabilizer muscles within the lumbo-pelvic-hip complex (e.g., transversus abdominis, lumbar multifidus). Individual worksite trials [24,25,26,35] have demonstrated that progressive Pilates routines can enhance trunk stability and localized endurance.

Modern workplaces impose diverse physical challenges that can compromise functional capacity. Sedentary employees experience strain from prolonged static sitting, healthcare workers face spinal shear stress during patient transfers, and military helicopter pilots endure whole-body vibrations and sustained asymmetric postures [35]. Deficits in core stability can compromise passive ligamentous structures and increase injury risk. While aggregated muscular endurance did not reach significance across these diverse cohorts, the targeted biomechanical adaptations fostered by Pilates align with the observed reductions in functional disability.

Occupational conditions such as chronic low back pain or upper cross syndrome limit functional capacity. Trials targeting upper-body impairments have reported improvements in functional markers like the Neck Disability Index [33]. Furthermore, long-term evaluations among healthcare professionals [36,37] suggest that improved spinal alignment and movement control translate into enhanced workplace performance. By restoring optimal length-tension relationships and movement quality, Pilates interventions appear to modify faulty movement patterns. This effectively reduces functional impairment caused by occupational demands, even if raw physiological endurance metrics vary among different worker populations.

### 4.4. The Pain–Disability Dissociation and the Fear-Avoidance Model

An important finding of this meta-analysis is the divergence between the non-significant reduction in musculoskeletal pain and the statistically significant improvement in functional disability. Contemporary rehabilitation literature recognizes that functional capacity can improve independently of changes in nociceptive intensity [45,46,47]. The Fear-Avoidance Model, as refined in modern clinical pain neuroscience [48,49] helps contextualize this dissociation, proposing that cognitive catastrophizing, kinesiophobia, and behavioral avoidance are primary drivers of disability.

Within occupational environments, employees experiencing persistent discomfort may develop somatic hypervigilance, misinterpreting benign mechanical sensations as indicators of tissue harm [50]. This cognitive distortion can lead to the avoidance of work-related physical tasks, ultimately contributing to muscular deconditioning, diminished functional capacity, and increased absenteeism [51]. In this context, Pilates, incorporating body awareness, controlled movement, and segmental alignment [52], functions as a structured form of graded exposure, enabling employees to safely navigate previously avoided ranges of motion. Supporting this mechanism, specific worksite trials, such as the one conducted by Taulaniemi et al. [36], have documented measurable reductions in fear-avoidance beliefs following Pilates interventions.

Compelling empirical evidence supports these mechanistic pathways: meta-analytic data demonstrate that Pilates interventions effectively attenuate kinesiophobia [53], while mediation analyses confirm that reductions in pain catastrophizing and kinesiophobia directly mediate functional capacity improvements following Pilates regimens [54]. Furthermore, targeted physical-cognitive workplace interventions have been shown to directly decrease work-related fear-avoidance beliefs [55], a finding echoed in specific occupational Pilates trials [56].

Consequently, pain self-efficacy, defined as the confidence to execute physical tasks despite ongoing discomfort [57], emerges as a central mechanism. As self-efficacy improves, employees learn to reinterpret somatic signals, sustaining their functional participation even when underlying pain levels remain static. This shift provides a plausible explanatory framework for the reduction in occupational disability observed in our pooled data, despite the lack of aggregate changes in subjective pain scores.

### 4.5. Clinical Implications and Therapeutic Paradigms

From a clinical perspective, the outcome patterns identified in this meta-analysis provide practical insights for occupational health physicians, physical therapists, and corporate ergonomists. The dissociation between absolute pain reduction and improved functional disability suggests a biopsychosocial mechanism, aligning with contemporary models of movement retraining and active rehabilitation [58]. Rather than operating purely as an analgesic modality, workplace Pilates appears to function primarily as an effective method for functional re-education.

Although aggregated data did not confirm uniform improvements in muscular endurance across all occupational sectors, the biomechanical principles of systematic core stabilization inherent to Pilates remain clinically relevant. These movement strategies are designed to counteract localized neuromuscular fatigue of the lumbo-pelvic-hip complex and cervical stabilizers, which are recognized contributors to postural collapse and cumulative spinal loading during prolonged static work [59].

Therefore, occupational clinicians should approach Pilates not solely with the objective of eliminating somatic pain, but as a strategic intervention to reduce kinesiophobia, optimize spinal mechanics, and enhance movement self-efficacy. This approach enables symptomatic employees to execute professional tasks with greater biomechanical efficiency, redirecting the therapeutic focus from passive symptom management toward active functional optimization in the workplace.

Furthermore, across all aggregate outcomes, the 95% prediction intervals extended across the line of no effect. While the overall point estimates suggest favorable functional trends, these wide prediction intervals underscore significant statistical uncertainty regarding expected treatment outcomes in future, individual workplace intervention settings.

### 4.6. Psychological Manifestations and Health-Related Quality of Life

Our analysis did not demonstrate a statistically significant pooled improvement in health-related quality of life, and this finding was accompanied by substantial statistical heterogeneity (*I*^2^ > 89.4%). Although Pilates is classified as a mind–body exercise that integrates breathing control and somatic mindfulness [60], the pooled data suggest that its psychological benefits in the workplace are not uniform. Modern corporate environments impose high cognitive loads and varying degrees of chronic stress.

While individual trials suggest that workplace exercise may reduce fatigue or provide cognitive distraction [61], the high variance in our quantitative synthesis indicates that psychological responses are context-dependent. It is possible that employees with elevated baseline stress exhibit measurable gains, whereas cohorts with different stress profiles demonstrate minimal changes. Furthermore, following the exclusion of confounding data, our pooled estimate relies on a severely limited number of independent cohorts (*k* = 2). Consequently, current evidence does not support a generalized improvement in quality of life across all working populations, highlighting the need to interpret these psychosocial outcomes cautiously.

### 4.7. Occupational and Economic Implications Across Workforce Sectors

Workplace Pilates interventions exhibit divergent functional outcomes depending on the biomechanical demands of various occupational sectors. This factor likely drives the substantial heterogeneity observed in the primary analyses. Office workers primarily face risks associated with sedentary behavior; in this group, trials such as those by Jiang et al. [33] reported localized improvements in mobility rather than broad systemic changes. Correspondingly, broader occupational metrics, such as generic work ability, often show minimal alteration [62] because office tasks remain predominantly cognitive. Conversely, in physically demanding professions such as healthcare, interventions target high mechanical loads and physical strain during patient handling, as demonstrated in trials with nursing personnel by Taulaniemi et al. [34].

Conversely, healthcare workers face heavy physical demands, where musculo-skeletal injuries directly impair job performance [63]. While previous long-term evaluations [33] have explored reductions in sickness absence, our aggregated data indicate that improvements in muscular endurance are not statistically uniform across these diverse cohorts. Furthermore, the inclusion of military helicopter pilots [35] expands the applicability of Pilates to high-stress operational environments characterized by mechanical flight vibrations. Additionally, variations in structural parameters, such as micro-breaks integrated within working hours versus longer, high-intensity sessions conducted off-duty, further contribute to the divergence in reported physical and psychosocial outcomes. However, rather than producing uniform improvements across all professions, the pooled data indicate that the most reliable benefit of workplace Pilates is the reduction of functional disability. Therefore, Pilates should be viewed as a context-dependent intervention whose efficacy relies on tailoring the program to the specific requirements and operational constraints of the workforce sector.

### 4.8. Workplace Feasibility and Administrative Considerations

Beyond clinical efficacy, the practical implementation and administrative feasibility of exercise protocols are important considerations for organizational stakeholders [64]. Mat-based Pilates interventions offer logistical advantages, requiring minimal specialized equipment and adaptable spatial configurations. Nevertheless, occupational workloads and time constraints are recognized barriers to employee adherence in corporate settings [65]. To optimize compliance, wellness programs must strategically schedule brief sessions around shifts or lunch intervals. Given the contemporary shift toward remote and hybrid work models, integrating tele-exercise Pilates protocols represents a scalable alternative.

Financially, while some preliminary narrative evidence has pointed toward decreased sickness absence [66], our quantitative synthesis urges a more cautious economic interpretation. The reduction in functional disability provides a plausible mechanism for mitigating the socio-economic burdens of presenteeism. However, the lack of statistically significant improvements in absolute pain intensity and overall quality of life dictates that explicit claims regarding a definitive return on investment (ROI) should be avoided until confirmed by large-scale, rigorously controlled trials.

### 4.9. Methodological Insights and Data Dependency

A critical methodological requirement of this meta-analysis was the careful management of data dependency. Several included reports originated from the same primary trials. For instance, three publications [34,36,37] were derived from a single randomized controlled trial involving 219 healthcare professionals. Furthermore, multiple effect sizes for outcomes such as muscular endurance and quality of life were initially reported from identical participant cohorts in other primary studies.

Treating these multiple reports or correlated outcomes as independent observations would violate the fundamental assumption of meta-analytic independence, artificially inflate sample sizes, and bias the pooled estimates. To address this unit-of-analysis issue, we employed outcome aggregation prior to synthesis. By computing a single composite effect size per independent study cohort, we maintained statistical integrity and provided a reliable representation of the available evidence.

### 4.10. Limitations and Future Directions

The findings of this quantitative synthesis must be interpreted cautiously due to methodological limitations. First, substantial statistical heterogeneity (*I*^2^ > 88%) was observed across most outcome measures. Importantly, the 95% prediction intervals computed for pain, muscular endurance, and quality of life cross the null value. This indicates high uncertainty, suggesting that future comparable interventions could plausibly yield negligible benefits or differing effects depending on the context. This observed heterogeneity likely originates from key clinical and programmatic variations across primary studies, including differences in participants’ baseline pain status (ranging from healthy, asymptomatic workers to employees with chronic non-specific symptoms), variations in occupational physical demands (sedentary office workers vs. healthcare personnel and military pilots), and distinct intervention formats (e.g., short workplace micro-breaks during working hours versus longer, off-duty structured exercise sessions). These programmatic and contextual differences directly impact the stability of the pooled estimates, thereby limiting the generalizability of the combined effect sizes across unstandardized workplace environments.

Second, following the aggregation of dependent data, the number of independent study cohorts per outcome category is small. This constraint reduces the statistical power of the analyses and limits the generalizability of the pooled estimates. Third, due to the nature of active exercise interventions, blinding of participants and instructors was unfeasible. This lack of blinding introduces an inherent risk of performance and response bias, which is particularly impactful when evaluating subjective, self-reported metrics such as pain intensity and quality of life. Future research in corporate wellness should prioritize rigorously controlled, sector-specific longitudinal trials to minimize these biases and clarify the true occupational efficacy of Pilates.

Furthermore, future trials should explicitly stratify and report outcomes based on participants’ baseline clinical status, differentiating between symptomatic cohorts receiving rehabilitative interventions and asymptomatic cohorts undergoing preventative programs, as this baseline variability appears to be a major driver of the observed statistical heterogeneity.

## 5. Conclusions

This meta-analysis indicates that workplace Pilates interventions consistently reduce functional disability among employees. However, pooled estimates for musculoskeletal pain intensity, muscular endurance, and health-related quality of life did not reach statistical significance, and these outcomes exhibited substantial statistical heterogeneity. These findings suggest that while Pilates may not uniformly improve all physical and psychosocial parameters across diverse occupational settings, it offers targeted benefits for functional capacity.

Given the variability across workplaces, intervention protocols, and measurement tools, these results should be interpreted cautiously. More standardized, multicenter randomized controlled trials with consistent reporting and objective outcome measures are needed to clarify the magnitude and durability of workplace Pilates effects.

## Figures and Tables

**Table 1:** Summary of Characteristics of the Included Studies (*Ν* = 12 Trials/14 Reports).

Author (Year)	Target Population	Sample Size (*N*)	Intervention Protocol	Outcomes Extracted
Sabir et al. [24]	Office Workers/Healthcare	46	Pilates vs. Control	Pain, Disability
Karkousha et al. [25]	Office Workers/Healthcare	40	Pilates vs. Control	Pain, QoL
Kolomiitseva et al. [26]	Office Workers/Healthcare	32	Pilates vs. Control	Endurance
Maan et al. [27]	Office Workers/Healthcare	52	Pilates vs. Control	Pain, Disability
Azam et al. [28]	Office Workers/Healthcare	30	Pilates vs. Control	QoL
Parang et al. [29]	Office Workers/Healthcare	30	Pilates vs. Control	Endurance, QoL
Alves et al. [30]	Office Workers/Healthcare	32	Pilates vs. Control	Pain, QoL
Dale et al. [31]	Office Workers/Healthcare	28	Pilates vs. Control	QoL, Endurance
Bulguroglu et al. [32]	Office Workers/Healthcare	58	Pilates vs. Control	Pain, Disability
Jiang et al. [33]	Office Workers (Neck/UCS)	30	Pilates vs. Control	VAS, NDI
Taulaniemi et al. [34]	Healthcare Personnel	219	Pilates vs. Control	FABs, Sickness absence
Bulhões et al. [35]	Helicopter Pilots	15	Pilates vs. Control	Pain, Disability, Endurance

Note: QoL, Quality of Life; VAS, Visual Analog Scale; NDI, Neck Disability Index; FABs, Fear-Avoidance Beliefs; UCS, Upper Cross Syndrome; Endurance refers to cervical or trunk muscular endurance as reported in each trial. The total number of reports (*Ν* = 14) includes two additional publications derived from the same randomized controlled trial among Finnish healthcare workers: Taulaniemi et al. [36] (secondary analysis on fear-avoidance beliefs) and Kolu et al. [37] (24-month follow-up analysis). These publications originate from the same parent trial and therefore do not appear separately in Table 1, which summarizes distinct RCTs only.

**Table 2:** Risk of Bias Assessment for Randomized Controlled Trials (Cochrane RoB 2 Tool).

Study (Author, Year)	D1	D2	D3	D4	D5	Overall Risk of Bias
Sabir et al. [24]	!	!	+	!	+	Some Concerns
Karkousha et al. [25]	+	+	+	+	+	Low Risk
Kolomiitseva et al. [26]	!	!	+	!	!	Some Concerns
Maan et al. [27]	+	!	+	+	+	Low Risk
Azam et al. [28]	!	!	+	!	!	Some Concerns
Parang et al. [29]	!	!	+	!	!	Some Concerns
Alves et al. [30]	!	!	+	!	+	Some Concerns
Dale et al. [31]	!	!	+	!	!	Some Concerns
Bulguroglu et al. [32]	+	!	+	+	+	Low Risk
Jiang et al. [33]	+	!	+	+	+	Low Risk
Taulaniemi et al. [34]	+	!	+	+	+	Low Risk
Bulhões et al. [35]	+	!	+	+	+	Low Risk

Notes: D1 = Randomization process; D2 = Deviations from intended interventions; D3 = Missing outcome data; D4 = Measurement of the outcome; D5 = Selection of the reported result. (+) = Low Risk; (!) = Some Concerns.

**Table 3:** Meta-Analysis Results: Musculoskeletal Pain Intensity.

Metric	Estimate	Standard Error (SE)	95% CI (Lower)	95% CI (Upper)	*p*-Value/Test
Pooled Effect (Hedges’ *g*)	−0.461	0.465	−1.599	0.677	*p* = 0.360 (*t* = −0.99)
Heterogeneity (*Q*)	48.83	-	-	-	*df* = 6, *p* < 0.001
Heterogeneity (*I*^2^)	93.03%	-	81.94%	98.87%	-
Between-Study Variance (tau^2^)	1.214	0.783	0.413	7.966	-
Between-Study Variance (tau)	1.102	0.355	0.642	2.822	-
95% Prediction Interval (*PI*)	-	-	−3.387	2.465	-

Note: CI, Confidence Interval; SE, Standard Error; *Q*, Cochran’s *Q* statistic; *I*^2^, percentage of total variability due to heterogeneity; tau^2^, between-study variance; tau, square root of tau^2^; PI, Prediction Interval. Estimates derived from an aggregated random-effects model using Hedges’ *g*.

**Table 4:** Meta-Analysis Results: Functional Disability.

Metric	Estimate	Standard Error (SE)	95% CI (Lower)	95% CI (Upper)	*p*-Value/Test
Pooled Effect (Hedges’ *g*)	−0.660	0.168	−1.091	−0.229	*p* = 0.011 (*t* = −3.94)
Heterogeneity (*Q*)	7.14				*df* = 5, *p* = 0.211
Heterogeneity (*I*^2^)	32.02%		0.00%	87.61%	
Between-Study Variance (tau^2^)	0.057	0.112	0.000	0.854	
Between-Study Variance (tau)	0.239	0.235	0.000	0.924	
95% Prediction Interval (PI)			−1.409	0.089	

Note: CI, Confidence Interval; SE, Standard Error; *Q*, Cochran’s *Q* statistic; *I*^2^, percentage of total variability due to heterogeneity; tau^2^, between-study variance; tau, square root of tau^2^; *PI*, Prediction Interval. Estimates derived from an aggregated random-effects model using Hedges’ *g*.

**Table 5:** Meta-Analysis Results: Muscle Endurance.

Metric	Estimate	Standard Error (SE)	95% CI (Lower)	95% CI (Upper)	*p*-Value/Test
Pooled Effect (Hedges’ *g*)	1.354	0.974	−1.150	3.857	*p* = 0.223 (t = 1.39)
Heterogeneity (*Q*)	104.57	-	-	-	*df* = 5, *p* < 0.001
Heterogeneity (*I*^2^)	97.54%	-	93.49%	99.61%	-
Between-Study Variance (tau^2^)	5.383	3.534	1.951	34.55	-
Between-Study Variance (tau)	2.320	0.762	1.397	5.878	-
95% Prediction Interval (PI)	-	-	−5.115	7.822	-

Note: CI, Confidence Interval; SE, Standard Error; *Q*, Cochran’s *Q* statistic; *I*^2^, percentage of total variability due to heterogeneity; tau^2^, between-study variance; tau, square root of tau^2^; PI, Prediction Interval. Estimates derived from an aggregated random-effects model using Hedges’ *g*.

**Table 6:** Meta-Analysis Results: Health-Related Quality of Life (QoL).

Metric	Estimate	Standard Error (SE)	95% *CI* (Lower)	95% CI (Upper)	*p*-Value/Test
Pooled Effect (Hedges’ *g*)	−0.146	0.487	−6.338	6.046	*p* = 0.815 (*t* = −0.30)
Heterogeneity (*Q*)	9.41	-	-	-	*df* = 1, *p* = 0.002
Heterogeneity (*I*^2^)	89.37%	-	46.61%	99.95%	-
Between-Study Variance (tau^2^)	0.425	0.672	0.044	100.0	-
Between-Study Variance (tau)	0.652	0.516	0.210	10.00	-
95% Prediction Interval (PI)	-	-	−10.491	10.190	-

Note: CI, Confidence Interval; *SE*, Standard Error; *Q*, Cochran’s *Q* statistic; *I*^2^, percentage of total variability due to heterogeneity; tau^2^, between-study variance; tau, square root of tau^2^; PI, Prediction Interval. Estimates derived from an aggregated random-effects model using Hedges’ *g*.

**Table 7:** GRADE Summary of Findings for Workplace Pilates Interventions.

Outcome	No. of Studies (*k*)	Risk of Bias	Inconsistency	Indirectness	Imprecision	Publication Bias	Certainty of Evidence (GRADE)	Key Findings/Effect Size
Functional Disability	6 RCTs (*n* = 188)	Serious	Not Serious (*I*^2^ = 32.0%)	Not Serious	Serious	Undetected	LOW	*g* = −0.660 (95% *CI*: −1.091 to −0.229, *p* = 0.011); *PI* includes null (−1.410 to 0.089)
Pain Intensity	7 RCTs (*n* = 366)	Serious	Very Serious (*I*^2^ = 93.0%)	Not Serious	Serious	Undetected	VERY LOW	*g* = −0.461 (95% *CI*: −1.599 to 0.677, *p* = 0.360); Wide *PI* (−3.387 to 2.465)
Muscular Endurance	6 RCTs (*n* = 291)	Serious	Very Serious (*I*^2^ = 97.5%)	Not Serious	Very Serious	Undetected	VERY LOW	*g* = 1.354 (95% *CI*: −1.150 to 3.857, *p* = 0.223); Wide *PI* (−5.115 to 7.822)
Health-Related QoL	2 RCTs (*n* = 170)	Serious	Very Serious (*I*^2^ = 89.4%)	Not Serious	Very Serious	Undetected	VERY LOW	*g* = −0.146 (95% *CI*: −6.338 to 6.046, *p* = 0.815); Wide *PI* (−10.491 to 10.190)

Note: Risk of Bias: Downgraded by 1 level due to performance and response bias inherent to non-blinded physical exercise interventions and subjective self-reported measures. Imprecision: Downgraded by 1 level due to small aggregated sample sizes and prediction intervals crossing the line of no effect. Inconsistency: Downgraded by 2 levels due to extreme statistical heterogeneity (*I*^2^ > 85%) across study cohorts. Imprecision (Very Serious): Downgraded by 2 levels due to very small total sample size (*k* ≤ 6 for most outcomes), extremely wide 95% confidence intervals, and wide prediction intervals.

## Data Availability

No new data were created or analyzed in this study. Data sharing is not applicable to this article.

## Supplementary Materials

The following supporting information can be downloaded at https://www.mdpi.com/article/10.3390/jfmk11030340/s1: File S1: Combined Supplementary Materials Document, including: Section S1: Detailed Electronic Search Strategies and Boolean Strings; Section S2: PRISMA 2020 Checklist; Section S3: Data Ex-traction Framework and Characteristics of Included Randomized Controlled Trials; Section S4: Casewise Diagnostics and Leave-One-Out Sensitivity Analyses.

## Abbreviations

The following abbreviations are used in this manuscript:

WMSDs	Work-related musculoskeletal disorders
QoL	Quality of Life
REML	Restricted Maximum Likelihood
VAS	Visual Analog Scale
NDI	Neck Disability Index
UCS	Upper Cross Syndrome
DFFITS	Difference in Fits
FABs	Fear-Avoidance Beliefs
