# Supplementary material for: Pilates in the Workplace: A Systematic Review and Meta-Analysis of Physical, Psychological, and Functional Outcomes in Employees
Source: J Funct Morphol Kinesiol. 2026 Aug 29;11(3):340. doi: 10.3390/jfmk11030340 (PMC13608647; doi:10.3390/jfmk11030340)
Supplement: Supplementary file 1 [file jfmk-11-00340-s001.zip › jfmk-4453599-supplementary.pdf]

SUPPLEMENTARY MATERIALS

Pilates in the Workplace: A Systematic Review and Meta-Analysis of Physical, Psychological, and Functional Outcomes in Employees.

This comprehensive supplementary document contains all the supporting sections (Sections S1–S4) for the manuscript as required for submission to MDPI. All text, descriptions, and data structures have been fully prepared in English.

SECTION S1: DETAILED ELECTRONIC SEARCH STRATEGIES AND BOOLEAN STRINGS

The exact Boolean search strings applied across the four electronic databases to identify relevant randomized controlled trials (RCTs) are detailed below:

PubMed (MEDLINE)

("Pilates Training" [MeSH Terms] OR "Pilates" [Title/Abstract] OR "Pilates-based" [Title/Abstract]) AND ("Workplace" [MeSH Terms] OR "Occupational Health" [MeSH Terms] OR "workplace" [Title/Abstract] OR "employee*" [Title/Abstract] OR "worker*" [Title/Abstract] OR "occupational" [Title/Abstract]) AND ("musculoskeletal pain" [Title/Abstract] OR "functional disability" [Title/Abstract] OR "muscle endurance" [Title/Abstract] OR "quality of life" [Title/Abstract]) AND ("Randomized Controlled Trial" [Publication Type] OR "randomized" [Title/Abstract] OR "RCT" [Title/Abstract])

Scopus

TITLE-ABS-KEY ("Pilates") AND TITLE-ABS-KEY ("workplace" OR "occupational health" OR "employee*" OR "worker*" OR "occupational") AND TITLE-ABS-KEY ("musculoskeletal pain" OR "functional disability" OR "quality of life" OR "endurance") AND TITLE-ABS-KEY ("randomized controlled trial" OR "RCT" OR "randomized")

Cochrane Library (CENTRAL)

(Pilates): ti, ab, kw AND (workplace OR "occupational health" OR employee* OR worker* OR occupational): ti,ab, kw AND ("musculoskeletal pain" OR "functional disability" OR "quality of life" OR endurance): ti, ab, kw

PEDro (Physiotherapy Evidence Database)

Abstract & Title: Pilates | Therapy: fitness training | Problem: pain | Subdiscipline: ergonomics or occupational health | Method: clinical trial

SECTION S2: PRISMA 2020 CHECKLIST

Mapping of items from the PRISMA 2020 statement to the corresponding locations within the manuscript:

**Section and Topic**	**Item #**	**Checklist Item**	**Location in Manuscript**
**TITLE**	1	Identify the report as a systematic review.	Title
**ABSTRACT**	2	See the PRISMA 2020 for Abstracts checklist.	Abstract
**INTRODUCTION**	3	Describe the rationale for the review in the context of existing knowledge.	Section 1. Introduction
	4	Provide an explicit statement of the objective(s) or question(s).	Abstract & Section 1
**METHODS**	5	Specify the inclusion and exclusion criteria.	Section 2.3 (PICOS)
	6	Specify all databases and information sources.	Section 2.2
	7	Present the full search strategies for all databases.	Section 2.2 & Supp. File 1
	8	Specify the methods used to decide study eligibility.	Sections 2.3 & 3.1
	9	Specify the methods used to collect data from reports.	Section 2.4
	10a	List and define all outcomes for which data were sought.	Section 2.3 (Outcomes)
	11	Specify the methods used to assess risk of bias.	Section 2.4 & 3.3
	13a	Describe processes used to decide synthesis eligibility.	Sections 2.3 & 2.5
	13b	Describe methods required to prepare the data.	Section 2.6

Section and Topic	Item #	Checklist Item	Location in Manuscript
	13c	Describe methods used to tabulate or visually display results.	Section 2.5 (Plots)
	13d	Describe methods used to synthesize results and rationale.	Section 2.5 (Random-effects)
	13e	Describe methods used to explore heterogeneity.	Section 2.5 & Supp. File 4
	14	Describe methods used to assess risk of bias due to missing results.	Section 2.5 (Publication bias)
**RESULTS**	16a	Describe the results of the search and selection process.	Section 3.1 & Figure 1
	17	Cite each included study and present its characteristics.	Section 3.2 & Table 1
	18	Present assessments of risk of bias for each included study.	Section 3.3 & Table 2
	20a	Summarise characteristics and risk of bias for syntheses.	Sections 3.2 & 3.3
	20b	Present results of all statistical syntheses conducted.	Section 3.4 (Tables 3-6)
	21	Present results of any investigations of heterogeneity.	Section 3.5
**DISCUSSION**	23a	Provide a general interpretation of the results.	Section 4. Discussion
	23b	Discuss any limitations of the evidence included.	Section 4 (Limitations)
	23c	Discuss any limitations of the review processes used.	Section 4 (Limitations)
	23d	Discuss implications for practice, policy, and research.	Section 4 & Section 5
**OTHER**	24a	Provide registration information (PROSPERO).	Section 2.1

Section and Topic	Item #	Checklist Item	Location in Manuscript
	27	Declare any competing interests of review authors.	End of manuscript

SECTION S3: DATA EXTRACTION FRAMEWORK AND CHARACTERISTICS OF INCLUDED RANDOMIZED CONTROLLED TRIALS

The following overview outlines the baseline and methodological architecture of the 12 included randomized controlled trials compiled from the data extraction repository:

Study ID	Target Occupational Cohort	Total N	Pilates Protocol Details	Extracted Outcome Categories
Sabir et al. (2024)	Power loom workers	46	Mat Pilates (4 Weeks, 3x/week)	Pain, Disability, Other
Karkousha et al. (2021)	Office Workers / Healthcare	40	Workplace Mat Pilates	Pain, Quality of Life (QOL)
Kolomiitseva et al. (2018)	Office workers	32	Workplace/Mat Pilates	Muscular Endurance
Maan et al. (2023)	Healthcare and desk workers	52	Mat/Workplace Pilates	Pain, Disability, Endurance
Azam et al. (2022)	Sedentary workers	30	Mat Pilates intervention	Pain, Quality of Life (QOL)
Parang et al. (2019)	Desk-bound professionals	30	Mat/Workplace Pilates	Muscular Endurance, QoL
Alves et al. (2020)	Sedentary employees	32	Workplace Pilates sessions	Pain, Endurance, QoL

Study ID	Target Occupational Cohort	Total N	Pilates Protocol Details	Extracted Outcome Categories
Dale et al. (2015)	Office employees	28	Workplace exercise program	Muscular Endurance, QoL
Bulguroglu et al. (2023)	Healthy sedentary individuals	58	Face-to-face Pilates protocol	Pain, Endurance, QoL
Jiang et al. (2022)	Office Workers (Neck/UCS)	30	Mat/Workplace Pilates	Pain (VAS), Disability (NDI)
Taulaniemi et al. (2019)	Healthcare Personnel with LBP	219	Neuromuscular/Pilates exercise	Disability, Pain
Bulhões et al. (2020)	Helicopter Pilots	15	Mat/Workplace Pilates	Pain, Disability, Endurance

SECTION S4: CASEWISE DIAGNOSTICS AND LEAVE-ONE-OUT SENSITIVITY ANALYSES

Overview

This section presents the final aggregated effect sizes (Hedges' g), variances, and standard errors for each study per outcome. These values were computed using outcome aggregation to resolve intra-study data dependency (multiple outcome measures reported for the same sample) prior to conducting the meta-analysis.

**Study**	**Outcome**	**Hedges' g**	**Variance**	**SE**	**N (Exp)**	**N (Ctrl)**	**Risk of Bias**
Alves et al.	Disability	-0.2279	0.1095	0.3309	20	17	Some Concerns
Dale et al.	Disability	-0.2856	0.1594	0.3992	8	9	Some Concerns
Jiang et al.	Disability	-0.9625	0.0934	0.3056	15	15	Some Concerns
Karkousha et al.	Disability	-0.3797	0.1362	0.3691	15	15	High Risk
Maan et al.	Disability	-0.7225	0.1438	0.3792	15	15	High Risk
Sabir et al.	Disability	-1.2157	0.1037	0.3220	22	22	High Risk
Alves et al.	Endurance	-0.7703	0.1168	0.3418	20	17	Some Concerns
Bulguroglu et al.	Endurance	5.7725	0.4079	0.6387	20	20	Low Risk / Some Concerns
Bulhões et al.	Endurance	1.8418	0.3809	0.6172	8	7	Low Risk
Kolomiitseva et al.	Endurance	1.7590	0.1690	0.4111	20	19	Some Concerns

Study	Outcome	Hedges' g	Variance	SE	N (Exp)	N (Ctrl)	Risk of Bias
Maan et al.	Endurance	-0.4682	0.1378	0.3712	15	15	High Risk
Taulaniemi et al.	Endurance	0.2443	0.0382	0.1953	64	66	Low Risk
Alves et al.	Pain	0.3507	0.1105	0.3324	20	17	Some Concerns
Azam et al.	Pain	0.7806	0.0615	0.2480	35	35	Some Concerns
Bulguroglu et al.	Pain	0.2863	0.1012	0.3181	20	20	Low Risk / Some Concerns
Bulhões et al.	Pain	-2.9394	0.5558	0.7455	8	7	Low Risk
Karkousha et al.	Pain	-1.4416	0.1802	0.4245	15	15	High Risk
Sabir et al.	Pain	-1.0031	0.0984	0.3137	22	22	High Risk
Taulaniemi et al.	Pain	-0.0090	0.0308	0.1755	64	66	Low Risk
Azam et al.	QoL	-0.6433	0.0601	0.2452	35	35	Some Concerns
Parang et al.	QoL	0.3316	0.0409	0.2022	50	50	Some Concerns
